# Dental Fees

**Published:** 1859-10

**Authors:** B. Wood

**Affiliations:** Dentist, Nashville


### DENTAL FEES.

BY B. WOOD, M. D., DENTIST.

This subject has received a good share of attention from time to time in our Journals. It merits all it has received, and I think, admits of further discussion without exhaustion. I propose some remarks relating to it, although I do not feel capable of doing it justice, and have not the time to arrange what I have to say in the best order for *print.*

It is conceded by all that the remuneration for dental services should be such as to command the requisite ability, and to compensate for bestowing all the care, labor and expense demanded for the successful execution of whatever we undertake. Competent and reliable operators, one would suppose, ought surely to receive *as much* for their *time* and *labor* as those who are not at all, or but illy qualified to discharge the duties they assume. Nevertheless it may be safely said as a general thing, the latter class obtain a larger remuneration for the actual amount of time and material expended than the former. Their patients too, pay the tax with cheerfulness, or even gratitude, imperfect or worthless as are the operations, while they would loudly complain if required by the former to pay a like sum for a similar amount of time and labor spent in the bestowment of substantial services.

I speak now of services belonging exclusively to the province of the dentist, and not of those pertaining in common to him and the physician or surgeon, which it is to be remarked, thanks to the medical profession, the skillful operator generally obtains better pay for the time occupied, than his unskilful competitor. In such cases the patient sometimes estimates the "services" by the amount of labor, as in the case where the regular fee for extraction was thought extortionate because another had *pulled at* the same tooth ten times as long,—dragging him to whom it was *attached*, all about the room,—and only charged half as much!

If a proficient operator would consent to plug or insert teeth upon the model of quackery, he could make, at *quack prices*, more than he could in the faithful execution of his task at even the highest rates. This is known but too well by those disposed to yield to popular prejudice in opposition to the dictates of conscience, preferring to put off inferior work at a reduced price, than to encounter the imputation of "extortion." The outward pressure sometimes compels to the adoption of this course as the only alternative to secure business. There are many places where the customary rates (as established by empiricism,) are insufficient to enable the practitioner to *learn* to execute well. Custom is of course inexorable, and he must remain in *statu quo* or retrograde, most probably the latter.

We say, no doubt, truly, the masses do not appreciate the importance of good operations, nor the skill required in their execution. Yet how often is it that the latter class, sufficiently affluent and intelligent, who know well enough to choose the good and eschew the bad, when they see unmistakeably, a reliable dentist has devoted vastly more time and labor (to say nothing of skill,) in a given case than is customary with pretenders, nevertheless demur at any thing like corresponding remuneration. We are frequently consulted in cases of bad fillings which the patients, conscious of their worthlessness, wish replaced, where it would have been easy to "stuff" a dozen or more after the same style in the time required to do any one of them perfectly; but should a dentist in such case charge twelve times the cost of the original plug, what a commotion would it stir up! Indeed he is often expected to do the work at "about the same." I have been more astonished at the stupidity, or audacity evinced in such instances, than any thing else I know of. It is remarkable that almost universally there appears to be little value attached to the *time* of dentists compared with the ostensible amount, or *number* of operations.

The evil of such a state of things is apparent—a premium for empiricism and dishonesty—a barrier to progress in the path of excellence.

Quackery has doubtless had much to do in bringing it about. Quackery holds the preponderance in point of numbers; is busy, active, untiring; everywhere at the right time, efficiently at work. Quackery is eloquent, persuasive, cogent in argument, and withal exceedingly sympathizing and philanthropic.

While the qualified dentist, with a proper respect for his calling, remains at his office relying upon diligently acquired knowledge and skill, the quack sallies abroad, is sociable, ingratiates himself with the dear people; "he talks as though he understood the business;" he "reasons well;" he offers to operate for them "*so* cheap"—at "Northern prices," at "Eastern prices," at "European prices," as the case may be. He tells them the charges of reliable dentists are extravagant, exorbitant, really an outrage. He explains how little gold it t·kes to plug a tooth, or put in one, and reasons mathematically of profit and loss. He wouldn't interest himself if it were only the rich; but persons moderate in circumstances can't stand it; and the poor—Ah, the poor! The quack is the especial sympathizer and guardian of the poor. He will take their money for pretended services which he knows to be worthless, and only calculated to lull them into a false security, until their teeth are lost and require a new set at his hands—at "half price;" at the same time conjuring up to their imaginations such spectres in regard to "extortionate charges" as effectually deter from seeking the counsel of those who are competent to benefit.

Nor is this confined altogether to quacks and pretenders. Educated but inexperienced dentists, and practitioners of fair reputation, but who have not attained the highest excellence, often join a like outcry against operators of deservedly higher eminence, which they will be sure to regret should they ever come to merit a like eminence; for they will find that it is only to be won and maintained at an expenditure of skill and labor which they have not as yet learned to bestow. To this may be added the culpable and mischievous propensity to disparage each other, incited by egotism, selfish ambition, cupidity or envy.

Beyond doubt, quackery, backed by such respectable aid, has exerted a wide-spread influence. But apart from this, there are other things which have perhaps equally contributed to the popular misconception and prejudice referred to, and prominent among these I am disposed to reckon the custom of charging by specific operations, rather than according to time occupied; thereby leading to misconception as to the basis of valuation. Thus in the operation of plugging teeth, the public have been led to affix a definite value to a plug, variable 'tis true, according to material used and according to size. But with them, in any given case, a plug is a plug, and they can't or won't comprehend why they should pay one operator more for the *same thing* than another. They acknowledge, 'tis true, a difference in the execution, and they learn that whereas the fillings of some stand and save the teeth, those of others do not. But they can't understand why it should *cost* much more for the former than for the latter; nay, why, as being less liable to need repair, they might not be afforded for less; conceiving at all events that the increase of business, sure to result from superior success, ought to be an ample reward.

That they should not be able to discriminate as to good and bad, even had they a fair inspection, is not strange, seeing that it takes a practical dentist to know a good plug from an indifferent one:—certain it is, some practitioners imagine their own to be very good, until time proves the contrary. It is folly to hope the masses will ever be competent to judge of the comparative excellence of our operations, although they can be taught to form some estimate as to the care and time bestowed upon them.

The number of plugs is no criterion of the extent or value of our services to a patient, since one series of operations may require thrice the time and material of another, the number of plugs being the same. Or in the same mouth the number of plugs may admit of multiplication or diminution. Thus in the crown of a molar, dotted with two or more decayed spots, all connected by seams or veins, one operator, influenced by low rates, might choose to introduce two, four, or more fillings, while another would feel it necessary to bring the whole into one, at an increased tax in time and material.

Similar references might be made to other operations; also to the putting up of artificial teeth. In the latter we all know how much labor can be saved, and yet the patient scarcely perceive the difference, although a faithful attention to all the details, which consume so large a proportion of time, makes a vast difference as to the perfection of the whole,—in harmonizing with the expression, in comfort and in general utility.

It will be seen upon how false a principle our customary estimate of fees is predicated, how it is calculated to mislead the public, what advantage it affords to empiricism and dishonesty, and how it serves to retard the march of excellence.

Another thing contributing to the popular prejudice with reference to remunerative fees, is the disreputable practice of *underbidding*. If a "job" is so greedily sought after that honorable men will stoop so low after it, the inference is fair that it must be a source of large emolument, and the lowest price thus bid will be set down as nearest its true value. I hope this sin is not common with recognized dentists. An honest shoemaker will scorn to underbid his neighbor, and his is a much more honorable calling than that whose regular members should be noted for such poor resorts. Difference in fees may and always will exist, but that is no extenuation for underworking one's own regular rates in order to get a "job" from a professional brother.

Allied to the foregoing is variableness in prices, according to customers—the "take what you can get" system. Now, as no one is expected to do work for less than he can afford, whatever he charges above his lower rates, provided the service or workmanship be the same, is regarded so much above par—in brief, a *shave*. But dealing with dentists every body negociates for the "best," and thinks he gets it; so that this deduction is sure to follow. Indeed, such a course usually marks out the *pretender* in the full sense of the word, whose lowest charges are generally all his "best" operations are worth. Nevertheless those unable to discriminate are thus misled to the general prejudice.

Another practice similar in its results, although in itself commendable if prompted by right motives, is that of dispensing services *in part* gratuitously:—services wholly gratuitous come so clearly under the head of benevolence that they need not give rise to misinterpretation. Other things the same, men are most liberal in that which costs little. A series of operations which would be speedily run over by the empiric at a trifling expense, would prove, in the hands of an honest and efficient dentist, a tedious and expensive task, and probably to the neglect of other business. It is therefore, hardly presumable that he should be over solicitous to do gratuitous work. When dentists not remarkable for benevolence in other things, are seen eager to undertake cases for the indigent or penurious at their own terms, it affords a fair presumption that it is attended with no sacrifice, and if the work is not slighted, which the plea of benevolence would seem to forbid, it seems to depreciate dental services in the same way as the instances above noticed: or if any thing is conceded on the score of generosity, it still argues such excessive rates in ordinary cases as shall compensate for the deficiency in these.

Those who have once received benefits in this way ever after expect the same, no matter how their circumstances may have improved. You charged me only so much before, is the standing appeal—(the bestowment of an *extra* fee in consideration of a former gratuity is unheard of). So also their acquaintances come with the like expectation. Howbeit they are willing the dentist should get what he can "out of the rich" whom they appear ready enough to turn over to him as legitimate subjects for *plunder*, provided only he will be "reasonable" with themselves. The liberal minded, on the contrary cannot see the justice of imposing an extra tax upon them for the benefit of others without so much as receiving credit for it. The effect upon the whole is prejudicial, resulting in general evil which probably far overshadows the good accomplished by partial gratuities.

No one would discourage acts of true generosity, but they should be clearly such, and bestowed with discrimination, especially where called for as a matter of ornament rather than utility. Many can afford artificial teeth when they can be got under price, who would feel too poor to spend half as much to preserve their natural teeth, though perhaps of infinitely more importance.

Having thus indicated some of the sources of popular prejudice and misconception in regard to dental fees, the corrections will readily suggest themselves:—a more correct standard of estimate for services, regulated by the time required for their efficient performance; greater uniformity of rates among the profession, and more scrupulous adherence to them by individual dentists.

NASHVILLE, Sept. 7th, 1859.

